# miR-503 represses human cell proliferation and directly targets the oncogene DDHD2 by non-canonical target pairing

**DOI:** 10.1186/s12864-015-1279-9

**Published:** 2015-02-05

**Authors:** Damon Polioudakis, Nathan S Abell, Vishwanath R Iyer

**Affiliations:** Department of Molecular Biosciences, Center for Systems and Synthetic Biology, Institute for Cellular and Molecular Biology, University of Texas, Austin, Texas USA

**Keywords:** miRNA, miRNA targeting, Proliferation, Ago2 immunoprecipitation, RIP-seq, miRNA targets, miRNA target pairing, miR-503, miRNA non-canonical pairing

## Abstract

**Background:**

The pathways regulating the transition of mammalian cells from quiescence to proliferation are mediated by multiple miRNAs. Despite significant improvements in our understanding of miRNA targeting, the majority of miRNA regulatory networks are still largely unknown and require experimental validation.

**Results:**

Here we identified miR-503, miR-103, and miR-494 as negative regulators of proliferation in primary human cells. We experimentally determined their genome wide target profiles using RNA-induced silencing complex (RISC) immunoprecipitations and gene expression profiling. Analysis of the genome wide target profiles revealed evidence of extensive regulation of gene expression through non-canonical target pairing by miR-503. We identified the proto-oncogene DDHD2 as a target of miR-503 that requires pairing outside of the canonical 5′ seed region of miR-503, representing a novel mode of miRNA-target pairing. Further bioinformatics analysis implicated miR-503 and DDHD2 in breast cancer tumorigenesis.

**Conclusions:**

Our results provide an extensive genome wide set of targets for miR-503, miR-103, and miR-494, and suggest that miR-503 may act as a tumor suppressor in breast cancer by its direct non-canonical targeting of DDHD2.

**Electronic supplementary material:**

The online version of this article (doi:10.1186/s12864-015-1279-9) contains supplementary material, which is available to authorized users.

## Introduction

The ability of human cells to transition from a quiescent to proliferative state is essential for tissue homeostasis. The majority of cells in an adult organism primarily exist in a quiescent state that is actively maintained by multiple genetic programs [1-3]. Maintenance of proliferative potential and the ability of cells to re-enter the cell cycle upon appropriate stimuli are vital for numerous physiological processes, including lymphocyte activation, hepatocyte regeneration, and wound healing [4-7]. The transition from quiescence to proliferation is exquisitely controlled by multiple check-points and fail-safe mechanisms, but is still susceptible to deregulation if the signaling pathways involved are sufficiently perturbed. Disruption of the actively maintained state of quiescence and sustained proliferative signaling are two of the basic requirements for tumorigenesis [8].

One of the main classes of regulators mediating cell proliferation and tumorigenesis are miRNAs [5,9]. miRNAs are short noncoding RNAs that post-transcriptionally regulate gene expression. They are ubiquitous regulators of multiple cellular pathways and biological processes such as metabolism, immune defense, development, differentiation, proliferation, and apoptosis [10,11]. miRNAs are predicted to regulate ~50% of all human protein coding genes [11], and have been shown to be involved in numerous pathologies, such as cardiovascular disease, viral diseases, and cancer development [12-14]. Multiple miRNAs are known to act as either tumor suppressors or oncogenes, and miRNAs are located in ~50% of all fragile regions in the genome or regions that are commonly amplified or deleted in cancers [15].

Genome wide gene expression profiling after miRNA perturbation, combined with sequence conservation analysis, has shown that efficacious miRNA-target pairing is mediated through perfect pairing of the seed region [12,16,17]. More recently, methodologies used to profile genome wide RISC occupancy have revealed extensive RISC binding to transcripts with imperfect or nonexistent seed matches [18-21]. This non-canonical miRNA-target pairing has been observed to occur in up to 60% of miRNA-target pairs [5,9,18]. However, regulation of gene expression by non-canonical target pairing was shown to be modest at best, and observed to have no significant effect on gene expression in multiple studies [12,16,17,20,21].

miR-503, miR-103, and miR-494 have previously been shown to act as either oncogenes or tumor suppressors in different cellular contexts [22-29]. miR-103 has been described to function as an oncomiR in colorectal cancer and breast cancer [22,27]. miR-494 has been reported to function as an oncomiR in colorectal cancer and non-small cell lung cancer [23,24], and as a tumor suppressor in cholangiocarcinoma and gastrointestinal stromal tumors [25,28]. miR-503 acts as a tumor suppressor in non-small cell lung cancer, glioblastoma, and hepatocellular carcinoma [26,30-33], and as an oncomiR in colon cancer [29]. Despite the broad involvement of miR-503 in repressing tumorigenesis in various cancers, its activity has not been explored in either primary cells or breast cancer.

In this study, we investigated the regulation of cell proliferation in primary human cells by miR-503, miR-103, and miR-494. We identified a genome wide set of the targets of miR-503, −103, and −494 by conducting extensive profiling of RISC associated transcripts and gene expression profiling. Analysis of the experimentally determined miRNA targetomes indicated utilization of non-canonical target pairing. We further explored the targeting mechanisms of miR-503 experimentally, and confirmed that miR-503 targets the proto-oncogene DDHD2 through a novel form of non-canonical target pairing. Additional bioinformatic analysis revealed a link between miR-503, DDHD2, and breast cancer.

## Results

### miR-503, miR-103, and miR-494 repress proliferation

miRNAs that regulate proliferation are differentially expressed in quiescent and proliferating primary human fibroblasts [34]. Using previously published data we identified numerous miRNAs induced in fibroblasts transitioning from quiescence to proliferation, and selected three, miR-503, miR-103, and miR-494, for further study that were consistently induced by serum stimulation and predicted to target proliferation and or cell cycle related genes [34].

**Figure 1:**
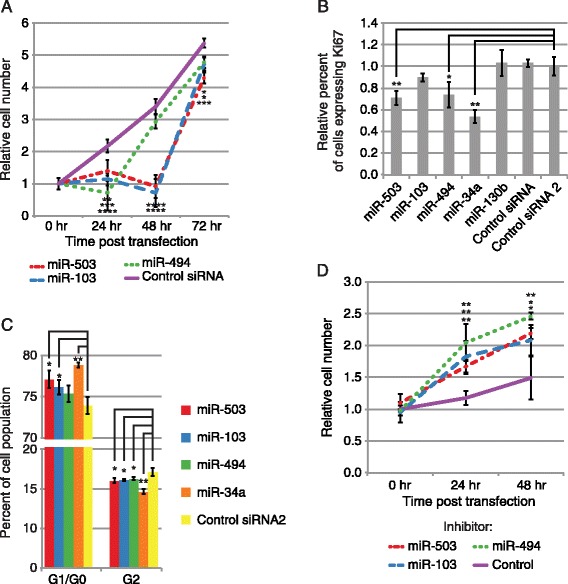
**miR-503, −103, and −494 inhibit proliferation. (A)** miR-503, −103, and −494 transfection significantly reduces primary human fibroblast cell growth. Average cell number relative to 0 hr following miRNA or Control siRNA transfection is shown for each time point indicated. Error bars denote ± SD, n = 6. P-values were calculated by Student’s t-test comparing cell numbers following miRNA transfection to cell numbers following Control siRNA transfection at each time point. **(B)** miR-503 and −494 transfection significantly represses proliferation. The Y-axis indicates the relative percentage of primary human fibroblast cells expressing Ki67, measured by flow cytometry. Bars are the mean percentage of cells expressing Ki67 relative to Control RNA 2, and error bars denote ± SD, n = 3. **(C)** miR-503 and −103 transfection significantly inhibits progression through the cell cycle. The Y-axis indicates the percentage of primary human fibroblast cells found in each stage of the cell cycle measured using propidium iodide staining, and bars are the mean percentage the cell population found in each stage. Error bars denote ± SD, n = 3. **(D)** Inhibition of miR-503, −103, and −494 increases cell growth in quiescent fibroblasts. Average cell number relative to 0 hr following transfection of an LNA targeting miR-503, −103, or −494 or a LNA negative control is shown for each time point indicated. Error bars denote ± SD, n = 6. P-values were calculated by Student’s one tailed t-test comparing cell numbers following miRNA inhibitor transfection to cell numbers following Control inhibitor transfection at each time point. For B, P-values were estimated by Student’s t-test, and for C, P-values were estimated by Student’s paired t-test. ****P < 0.0001, ***P < 0.001, **P < 0.01, *P < 0.05.

To investigate the effect of miR-503, miR-103, and miR-494 on cell proliferation, we transiently overexpressed each of these miRNAs in proliferating fibroblasts and assayed the rate of cell growth. Surprisingly, cells with transiently overexpressed miR-503, −103, or −494 showed significant reductions in the rate of cell growth (Figure 1A). miRNAs induced by serum stimulation were expected to increase proliferation, but there are numerous examples of miRNAs that participate in negative feedback loops [34-36]. To confirm their effect on cell growth, we overexpressed miR-503, −103, −494, and miR-34a, a well known tumor suppressor [37], in proliferating fibroblasts and counted cells expressing Ki67 protein using flow cytometry. miR-503 and −494 transient overexpression significantly decreased the percentage of Ki67 positive cells compared to multiple controls, indicating that these miRNAs repress proliferation, although not to the extent of miR-34a (Figure 1B) [37]. In addition, transient overexpression of miR-503 and −103 in proliferating fibroblasts significantly decreased the rate of progression through the cell cycle (Figure 1C). To rule out indirect effects from flooding the cells and the RNA silencing machinery with large quantities of the mature miRNA duplexes, we transiently inhibited miR-503, −103, and −494 in fibroblasts induced into quiescence by serum removal. Inhibition of miR-503, −103, and −494 in quiescent fibroblasts significantly increased the rate of cell growth (Figure 1D). Taken together, these data indicated that all 3 of these miRNAs inhibit cell growth, with miR-503 most clearly regulating proliferation and cell cycle progression.

### Genome wide profiling of miR-503, −103, and −494 targets

**Figure 2:**
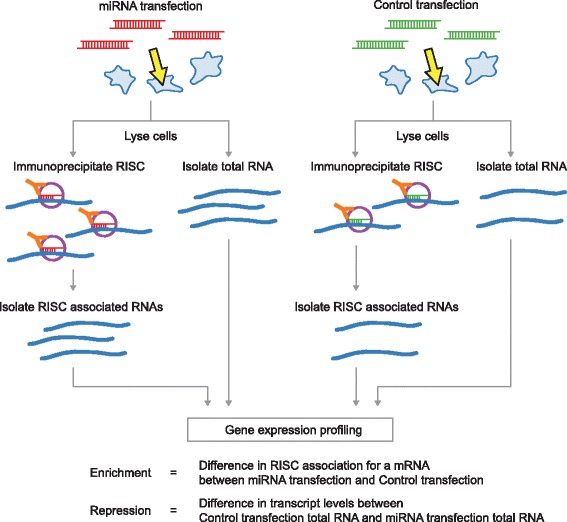
**Strategy for genome-wide target identification of miRNAs using RIP and gene expression profiling.** HeLa cells are transfected either with miRNAs or control duplexes. From each transfection, total RNA as well as RISC-associated RNAs are isolated by Ago2 immunoprecipitation. All populations of RNAs are profiled by deep sequencing or in come cases, by microarray hybridization (see text). RNAs showing RISC-association in a miRNA dependent manner are identified by comparing the immunoprecipitated RNAs between the miRNA and control RNA transfection. mRNAs showing repression by the miRNAs are identified by comparing the total RNAs between the miRNA and control RNA transfection.

To experimentally identify the targets of miR-503, −103, and −494 we used two approaches. First, we identified transcripts isolated from RISC immunoprecipitations (RIPs) following transient overexpression of each of the three miRNAs, using microarrays (RIP-chip) or deep sequencing (RIP-seq) (Figure 2). To immunoprecipitate RISC, we used a monoclonal antibody directed against Argonaute-2, an essential component of RISC [16]. RISC is directed to target mRNAs by the mature miRNA guide strand [16]. Increases in RISC association with a given transcript following miRNA overexpression indicate direct targets of the overexpressed miRNA. Second, we carried out gene expression profiling after transient overexpression of the miRNAs (Figure 2). RNA-Seq and microarray experiments were well correlated for both RIP and gene expression (Additional file 1). RIP enrichment was defined as an increase in RISC association with a given transcript following miRNA transfection compared to the control transfection. Enrichment indicates mRNAs associated with RISC in an miRNA dependent manner. To obtain enough RNA for successful genome wide profiling, all RIPs and gene expression experiments were conducted in HeLa cells, due to HeLa cells having a greater RNA content and being of a smaller size than human fibroblasts.

**Figure 3:**
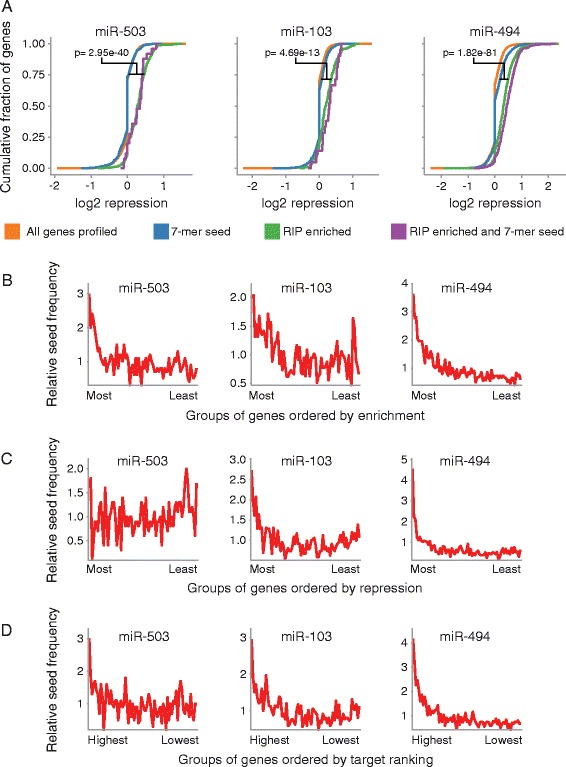
**Genome wide profiling of miR-503, −103, and −494 targets. (A)** mRNAs enriched in the RIP-seq following miR-503, −103, or −494 transfection are also repressed. The Y-axis denotes the cumulative fraction of all mRNA transcripts profiled for each group of mRNAs denoted by line color, and the X-axis indicates the level of repression for each mRNA transcript profiled with positive values indicating increased repression. Orange: all mRNAs; Blue: mRNAs that contain the respective 7-mer miRNA seed match in their 3′UTR; Green: mRNAs that were 1.75 fold enriched in the RIP; Purple: mRNAs that were 1.75 fold enriched in the RIP and that contain the respective 7-mer miRNA seed match in their 3′UTR. Significance estimates were calculated with Student’s t-test. **(B)** mRNAs enriched in the RIPs had the highest frequency of the respective miRNA seed matches. The X-axis denotes consecutive groups of 250 genes, ranked from most enriched to least enriched in the RIP-seq. **(C)** For miR-103, and −494, mRNAs repressed in the gene expression experiments had the highest frequency of the respective miRNA seed matches. The X-axis indicates consecutive groups of 250 genes, ranked from most repressed to least repressed. **(D)** The highest ranked miRNA targets had the highest frequency of the respective miRNA seed matches. RIP enrichment and gene expression repression data were combined to rank miRNA targets. The X-axis denotes consecutive groups of 250 genes, from most highly ranked to least highly ranked. For B, C, and D, The line is the frequency indicated on the Y-axis of the respective 7-mer miRNA seed in the gene group compared to the frequency of the seed in all genes profiled. For B, C, and D, n = 3. E combines gene expression data, n = 3, with RIP-seq data, n = 3.

To determine if the RIPs effectively identified miRNA targets, we first examined whether miRNA dependent mRNA association with RISC was accompanied by corresponding decreases in transcript levels. For all mRNAs profiled, the level of RISC association correlated well with repression of gene expression (Pearson correlation between RIP-seq enrichment and RNA-seq gene expression repression was 0.65, 0.63, and 0.58 for miR-503, −103, and −494 respectively). In addition, mRNAs that displayed miRNA dependent RISC association were significantly more repressed than all mRNAs profiled (Figure 3A). mRNAs that associated with RISC in a miRNA dependent manner were also significantly more repressed than all mRNAs containing the respective 7-mer miRNA seed match in their 3′UTR, indicating the RIPs more successfully identified mRNA targets than using presence of the seed match as the only criteria (Figure 3A).

As an additional measure of the effectiveness of our miRNA target identification, we examined miRNA seed match frequency in mRNAs associated with RISC in a miRNA dependent manner. Following transient miR-503, −103, and −494 overexpression, mRNAs with the greatest RISC association had a high frequency of the corresponding miRNA seed matches (Figure 3B). In addition, mRNAs that were most repressed following miRNA transient overexpression showed the greatest enrichment for miR-103, and −494 seed matches, although not for miR-503 (Figure 3C). Using a combination of both RIP enrichment data and repression of gene expression data to identify miRNA targets produced a high frequency of seed matches in the most highly ranked targets for all three miRNAs, and we therefore used this method to define our target sets (Figure 3D). There was enrichment for proliferation related genes in the targets identified for miR-503 and miR-494, but there were not enough target genes identified for miR-103 to produce meaningful results (Additional file 2) [38-40].

### Enrichment of sequences pairing to the 3′ end of the miRNAs in the experimentally profiled miRNA targetome

**Figure 4:**
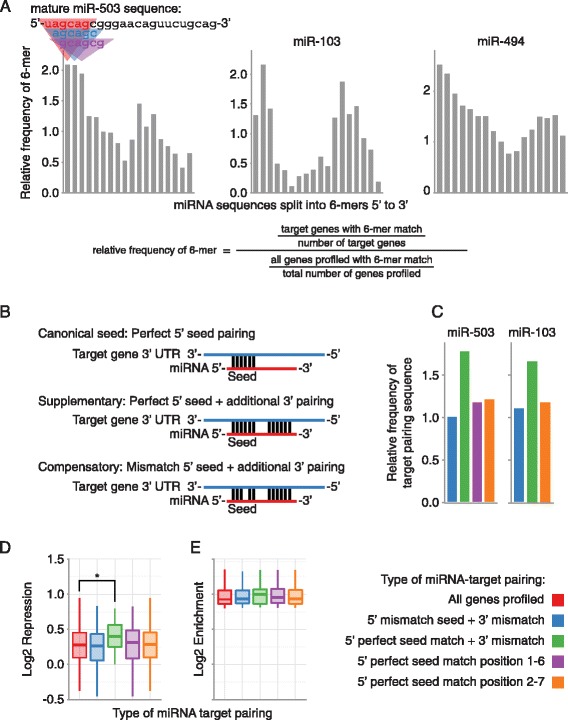
**miRNA targeting utilizing pairing outside of the canonical miRNA 5′ seed region. (A)** Enrichment of sequences pairing to the 3′ end of the miRNAs in the experimentally determined miRNA targetomes. The Y-axis indicates the frequency of a 6-mer in the experimentally determined miRNA target mRNAs relative to all mRNAs profiled. 6-mers are organized step-wise in 1 nucleotide increments along the X-axis from 5′ end to 3′ end of the mature miRNA. **(B)** Different types of miRNA-target pairing. **(C)** Frequency of different types of miRNA pairing in RIP-Seq enriched mRNAs. Bars are the frequency of mRNAs with the indicated type of miRNA-target pairing in the RIP enriched mRNAs, relative to the frequency of mRNAs with the indicated type of miRNA-target pairing in all mRNAs profiled. **(D)** RIP-seq enriched mRNAs containing a supplementary target site in their 3′UTR were significantly more repressed than all RIP-seq enriched mRNAs, but not more repressed than RIP-seq enriched mRNAs that contain a perfect 5′ seed in their 3′UTR (p = 0.04 and 0.06, respectively, Student’s t-test). Each box and whiskers plot indicates gene expression repression for genes that contain the type of miRNA-target pairing specified on the X-axis. Boxes extend from the 1^st^ to 3^rd^ quartile of gene expression repression, the band is the median, and whiskers denote the minimum and maximum excluding outliers. **(E)** There were no significant differences in miR-503 RIP enrichment in RIP enriched mRNAs with different types of miRNA-target pairing. Each box and whiskers plot indicates RIP enrichment for mRNAs that contain the type of miRNA-target pairing site specified on the X-axis. For C, D, and E, 5′ mismatch seed + 3′ mismatch (blue) corresponds to Compensatory in B; 5′ perfect seed match + 3′ mismatch (green) corresponds to Supplementary in B; and 5′ perfect seed match (purple and orange) correspond to Canonical seed in B. *P < 0.05.

To determine the sub-sequences of miR-503, −103, and −494 that are used for target pairing, we examined enrichment of 6-mer sequences that pair different areas of the mature miRNAs in the 3′UTRs of the experimentally identified targets (Figure 4A). As expected, there was a high frequency of 6-mer sequences that pair to the seed region, positions 2–7, of the mature miRNAs (Figure 4A). Surprisingly, there was also a high frequency of sequences that pair the 3′ end of the mature miRNAs (Figure 4A). In separately conducted experiments, we observed the same phenomenon in targets identified by RIP-Chip and microarray gene expression profiling (Additional file 3). Nelson et al. previously reported this same finding when they experimentally profiled the targets of miR-103, and concluded that miR-103 utilized miRNA 3′ pairing for targeting, although they did not further explore this observation experimentally [41]. Based on the enrichment for sequences pairing the miRNA 5′ seed and the miRNA 3′ end for miR-503 and miR-103, we hypothesized that miR-503 and −103 were using supplementary or compensatory pairing to target mRNAs (Figure 4B). Supplementary pairing employs a perfect miRNA 5′ seed match with additional 3′ pairing, and compensatory pairing uses additional 3′ pairing to compensate for mismatches or bulges in the 5′ seed match (Figure 4B)[16]. Although miR-494 targets also displayed extensive enrichment for 6-mers pairing outside of the miRNA 5′ seed region, the areas of enrichment were more diffuse, making them more difficult to investigate experimentally (Figure 4A).

To investigate the type of target pairing used by miR-503 and −103, we began by further analyzing our RIP and gene expression data. We first examined the frequency of compensatory and supplementary pairing sites in highly ranked targets. Since the most enriched 5′ 6-mer for miR-503 was from position 1–6 (Figure 4A), for miR-503 we used both a canonical 5′ position 2–7 seed match, as well as a 5′ position 1–6 seed match. The frequency of compensatory pairing sites in the target gene set for either miR-503 or miR-103 was not higher than the frequency of 5′ seed matches (Figure 4C & Additional file 3). However, supplementary pairing sites were much more frequent than the 5′ seed matches and compensatory pairing sites for both miR-503 and miR-103 (Figure 4C & Additional file 3).

Next we examined miRNA dependent RISC association and repression of mRNA targets with compensatory or supplementary pairing sites. mRNAs showing high miR-503 dependent RISC association and containing supplementary pairing sites were significantly more repressed than mRNAs showing only miR-503 dependent RISC association (Figure 4D). There were no significant differences in repression for miR-103 or in RISC association for either miR-503 or miR-103 that was dependent on pairing type (Figure 4E & Additional file 3). Taken together, this data strongly suggests miR-503 may utilize supplementary target pairing for repression. There were no differences between compensatory target pairing sites and the intact 5′ seed alone for both miR-503 and miR-103, making it difficult to draw conclusions as to utilization of compensatory target pairing.

### Pairing of the 3′ and 5′ end of miR-503 is necessary for direct targeting by miR-503 of the cell growth promoting gene DDHD2

**Figure 5:**
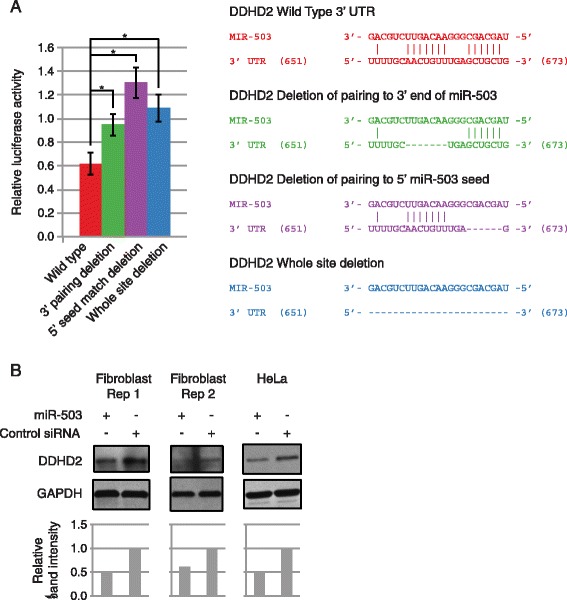
**Pairing of the 3′ and 5′ end of miR-503 is necessary for direct targeting by miR-503 of the proto-oncogene DDHD2. (A)** Pairing by the 3′ and 5′ end of miR-503 to the 3′UTR of DDHD2 is necessary to repress expression of the luciferase reporter construct. The Y-axis denotes relative luciferase units from miR-503 transfected HEK293 cells normalized to control RNA transfected cells. The X-axis indicates the type of 3′UTR (either wild-type or deletion as indicated) Error bars denote ± SD, n = 3. In the diagram, vertical lines indicate base pairing, dashes show deleted base pairs, and numbers in parenthesis denote location in the 3′ UTR, not genomic coordinates. P-values were calculated by Student’s one tailed t-test comparing miRNA normalized to Control siRNA luciferase activity with intact 3′ UTRs, to normalized activity with the respective miRNA target site deleted 3′ UTRs. *P < 0.05. **(B)** miR-503 transfection decreased protein expression of DDHD2. DDHD2 protein levels following miR-503 transfection in fibroblasts and HeLa cells compared to Control siRNA transfection. Band intensities were quantified, normalized to GAPDH, and shown relative to the Control siRNA.

To further investigate the utilization of supplementary target pairing by miR-503 in targeting genes that affected cell proliferation, we searched for candidate genes to test experimentally based on three criteria: (1) sequences in the 3′ UTR that paired the 5′ and 3′ end of miR-503 (Figure 5), (2) involvement in promoting cell proliferation, and (3) combined RIP-seq and RNA-seq gene expression repression rank. We identified one candidate gene, DDHD2, that contained a perfect miR-503 5′ seed match with additional 3′ pairing, had been previously shown to promote proliferation of breast cancer cells [42], and was the highest ranked gene in the combined genome-wide experiments that satisfied the first two criteria.

To experimentally determine the target pairing mechanisms used by miR-503, we cloned the 3′UTR of DDHD2 into luciferase reporter constructs, and constructed additional reporter constructs of the 3′UTR of DDHD2 with deletions of either the sequence pairing the 5′ end of miR-503, the sequence pairing the 3′ end of miR-503, or both (Figure 5A). miR-503 repressed expression of the reporter with the intact 3′UTR of DDHD2, and deleting the sequences pairing to the 5′ and 3′ portion of miR-503 relieved repression, indicating that miR-503 functionally targets DDHD2 for repression through these sites. Unexpectedly, deleting only the sequence pairing the 3′ end of miR-503 also significantly relieved repression by miR-503 even though the 5′ seed remained intact, indicating that miR-503 requires pairing of its 3′ end in order to target DDHD2 in addition to the 5′ perfect seed pairing (Figure 5A). Deleting the sequence pairing to the 5′ seed of miR-503 also significantly relieved repression, although there was no significant difference in reporter activity between the 3′ and 5′ deletion constructs (Figure 5A). To further confirm functional targeting of DDHD2 by miR-503, we transiently overexpressed miR-503 in proliferating fibroblasts and HeLa cells. Transient overexpression of miR-503 did not repress the transcript levels of DDHD2 in proliferating primary human fibroblasts (data not shown) but did decrease protein levels in both fibroblasts and HeLa cells, indicating miR-503 more strongly represses DDHD2 expression through inhibition of translation than mRNA destabilization (Figure 5B).

### The involvement of miR-503 in ER+ breast cancer

DDHD2 has previously been shown to be tumor promoting in breast cancer by cooperating functionally with Myc to stimulate breast cancer cell growth [42]. In addition, DDHD2 was observed to be located in a region (8p11.23) of high level copy number aberrations in breast tumors [42]. Amplification of this area most frequently occurs in estrogen receptor positive (ER+) breast tumors [42].

**Figure 6:**
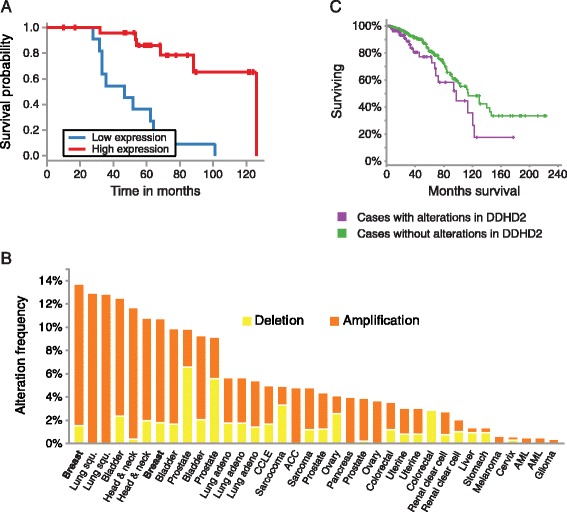
**miR-503 involvement in breast cancer. (A)** Low miR-503 expression correlates with a lower survival probability in ER+ breast cancer patients (p = 8.56e-06). Kaplan-Meier survival curves for patients with ER+ breast cancer expressing different levels of miR-503 were calculated using the MIRUMIR tool. Number of patients: n = 37. **(B)** DDHD2 is frequently amplified in breast cancer. The bars are the frequency of DDHD2 copy number alterations in the different types of cancer indicated on the X-axis. The frequency of patients with DDHD2 amplification was calculated using the cBio portal. Yellow: Deletion; Orange: Amplification. n = 1044. **(C)** Patients with copy number alterations in DDHD2 (purple line) have a lower survival probably than those without amplifications in DDHD2 (green line) (p = 0.027). Kaplan-Meier survival curves were was calculated using the cBio portal. n = 888.

We used the MIRUMIR tool to query the prognostic value of miR-503 expression levels in a published data set of patients with high-risk ER+ breast cancers [43]. Patients with high miR-503 expression had a significantly higher survival probability than patients with low miR-503 expression (Figure 6A). Analysis of DDHD2 alterations using the cBio portal revealed that DDHD2 tends to be amplified in a variety of cancers, and most predominantly in breast invasive carcinoma (~12% of cases) (Figure 6B). In addition, patients with copy number alterations of DDHD2 had a significantly lower survival probability than patients without copy number alterations of DDHD2 (Figure 6C). This data suggests miR-503 plays a role in breast cancer tumorigenesis at least in part by targeting DDHD2.

## Discussion

In this study, we experimentally determined the targetomes of multiple miRNAs that repress cell proliferation, and showed that miR-503 regulates the expression of the proto-oncogene DDHD2 by a unique non-canonical targeting mode. While supplementary base-pairing outside the 5′ seed has been noted before, to our knowledge, this is the first time that a requirement for supplementary pairing has been shown for target repression even when perfect complementarity in the canonical 5′ seed exists. We began by screening for miRNA involvement in primary cell proliferation, and identified miR-503, −103, and −494 as regulators of primary cell proliferation. We then constructed genome wide target sets for miR-503, −103, and −494 using RIP and global gene expression profiling methodologies. Our analysis of the target sets revealed potentially widespread use of non-canonical targeting by the miRNAs to post transcriptionally regulate gene expression. We went on to show that miR-503 targets the oncogene DDHD2 non-canonically, requiring 3′ pairing of miR-503 in addition to the normal 5′ pairing. Our analysis further linked miR-503 to ER+ breast cancer through DDHD2.

Recent advances in methodologies to profile the entire miRNA targetome have uncovered extensive mediation of RISC-mRNA association by non-canonical targeting [18-21]. However, the effects of non-canonical targeting on gene expression ranged from moderate at best, to statistically insignificant depending on the study [18,20,21]. This discrepancy may be due to differences in the reliance on miRNA seed interactions to identify targets. In our experiments, we found evidence of extensive non-canonical targeting by miR-503 mediating both RISC-target association and gene expression. We went on to experimentally confirm that 3′ target pairing was necessary for miR-503 to repress DDHD2. miR-503 targeting of DDHD2 may represent an isolated case, but analysis of our experimentally identified genome wide target set suggests miR-503 uses 3′ target pairing to mediate gene expression throughout its targetome.

In addition, we showed that miR-503 acts like a tumor suppressor by non-canonically targeting the putative oncogene DDHD2. DDHD2 was previously shown to promote tumorigenesis in breast cancer by cooperating functionally with Myc to enhance breast cancer cell growth [42]. DDHD2 is located in a region of the genome that is frequently amplified in breast tumors, and most frequently in ER+ breast tumors [42]. Although the exact area amplified differs between tumors, several other genes are located in the region and frequently amplified in addition to DDHD2. However, of this group of genes, only FGFR1 and DDHD2 were shown to function as oncogenes in breast cancer, and DDHD2 was shown to have transforming potential that was not dependent on FGFR1 expression [42]. Our analysis showed that decreased miR-503 expression correlated with low patient survivability, and that DDHD2 copy number amplifications were most frequent in invasive breast carcinoma and correlate significantly with low patient survivability. Taken together, these data suggest that miR-503 may function as a previously unknown tumor suppressor in ER+ breast cancer, and that a critical component of its tumor suppressor role could be the repression of DDHD2 via a non-canonical mechanism.

miR-503 has been shown to act as a tumor suppressor in several cancers, and decreases in miR-503 expression have been reported in multiple cancer types [26,30,32,44]. In addition, miR-503 has been shown to target multiple oncogenes and regulators of proliferation [30,33,44,45]. miR-503 inhibits hepatocellular carcinoma, glioblastoma multiforme, and non small cell lung cancer (NSCLC) tumorigenesis by repressing proliferation, inducing cell cycle arrest, and reducing metastasis [26,30,32,46]. Additionally, miR-503 also represses hepatocellular carcinoma tumor angiogenesis by targeting the potent angiogenic factors FGF2 and VEGFA [32]. miR-503 has also been shown to play a role in repressing endometrial cancer [44]. Considered as a whole, these studies suggest that miR-503 operates as a potent tumor suppressor that regulates multiple pathways that are frequently deregulated in tumorigenesis. Moderate repression of multiple key regulatory genes can have large phenotypic effects in concert, and miR-503 may conceivably inhibit breast cancer tumorigenesis by repressing multiple previously known targets in addition to DDHD2.

## Conclusions

In summary, we demonstrated that miR-503 represses cell proliferation, and may function as a tumor suppressor in ER+ cancer by utilizing non-canonical 3′ pairing to target and repress the proto-oncogene DDHD2. In addition, we provided experimentally determined genome wide target profiles of the proliferation related miRNAs miR-503, −103, and −494. Our genomic approach to identifying the targetome of miRNAs in combination with potential widespread use of non-canonical targeting mechanisms points to a broader role for several miRNAs in mediating cellular processes.

## Materials and Methods

### Normal cell culture conditions

HeLa cells and human foreskin fibroblasts (ATCC CRL #2091) were maintained in DMEM (Dulbecco’s Modified Eagle’s Medium) supplemented with 10% FBS (Fetal Bovine Serum) at 37°C under 5% CO_2_.

### RNA oligos and transfections

miRNA guide and anti-guide mature sequences were obtained from miRBase (http://www.mirbase.org/). Sequences for the siRNA against GFP (Control siRNA) were obtained from [47]; guide: 5′Phos-CUGGAGUUGUCCCAAUUCCUU and anti-guide: 5′Phos-AGAAUUGGGACAACUCCAGUU. Sequences for a scrambled control siRNA (Control siRNA 2) were adapted from [48]; 5′Phos-AAUUCUCCGAACGUGUCACGUUA and 5′Phos-ACGUGACACGUUCGGAGAAUUCA. RNA oligos were ordered from IDT and corresponding oligos were annealed in RNA annealing buffer (20 mM HEPES, pH 7.3, 50 mM KCl, 2 mM MgCl_2_) to form mature miRNA or siRNA duplexes. Both the miRNA and Control siRNA oligos contained 5′ phosphate and 3′ OH ends. The RNA duplexes were transfected at a final concentration of 100 nM using Lipofectamine 2000 according to the manufacturer’s instructions. The miRNA inhibitors and control were miRCURY Locked Nucleic Acid (LNA) miRNA Inhibitors and Negative Control Inhibitor obtained from Exiqon and transfected at a final concentration of 10 nM using Lipofectamine 2000.

### Cell counting assays

For miRNA overexpression experiments, fibroblasts were seeded at 10,000 cells per well in 6-well plates. Cells were cultured in DMEM supplemented with 10% FBS. Cells were allowed to grow 24 hours, and then cells were transiently transfected with miRNA or Control siRNA duplexes (100 nM final concentration). 0, 24, 48, and 72 hours after transfection, cells were trypsinized, and counted in a hemocytometer. 9 fields were averaged for each biological replicate. For miRNA inhibition experiments, fibroblasts were seeded at 10,000 cells per well in 6-well plates. Cells were cultured in DMEM supplemented with 10% FBS. Cells were allowed to grow 24 hours, and then media was replaced with low serum DMEM 0.1% FBS. 24 hours after replacement with DMEM 0.1% FBS, cells were transiently transfected with miRCURY Locked Nucleic Acid (LNA) miRNA Inhibitors or Negative Control Inhibitor obtained from Exiqon (10 nM final concentration). 0, 24, and 48 hours after transfection, cells were trypsinized, and counted in a hemocytometer. 9 fields were averaged for each biological replicate.

### Flow cytometry

Fibroblasts were seeded at 50,000 cells per well in 6-well plates. Cells were cultured in DMEM supplemented with 10% FBS. Cells were allowed to grow 24 hours, and then cells were transiently transfected with miRNA, Control siRNA, or Control siRNA 2 duplexes (100 nM final concentration). 24 hours after transfection, cells were trypsinized, washed with PBS, and fixed for 24 hours in 70% ethanol at −20°C. For Ki67 staining, after ethanol fixation, cells were washed with Stain Buffer (BD Pharmingen), incubated 30 minutes with FITC Mouse Anti-Human Ki67 antibody (BD Pharmingen), washed, and resuspended in 500 μl Stain Buffer with Propidium Iodide Staining Solution (5 μg/ml) (BD Pharmingen). For PI staining, after ethanol fixation, cells were washed twice with PBS, and resuspended in PI staining solution (50ug/mL PI with 100ug/mL RNase in PBS). Flow cytometry analysis for Ki67 in primary human fibroblasts was done using a FACsCalibur flow cytometer and 10,000 events above threshold levels were counted for each sample (BD Biosciences). Data analysis was done using FlowJo.

### RISC immunoprecipitation

We used a protocol previously detailed in [34] with some modifications. HeLa cells were grown in 10 cm^2^ tissue culture plates and transfected with either miRNA mature duplexes or Control siRNA duplexes at a final concentration of 100 nM or mock transfected. After 24 hours, cells were washed twice with PBS, and 0.5 ml of lysis buffer was added onto the cell monolayer followed by incubation at 4°C for 30 minutes. Cell lysate was collected by scraping and cleared by centrifugation at 14,000 rpm at 4°C. Cleared lysate was then incubated with 50 μl of protein-G beads (Roche) for 1 hour at 4°C (pre-clearing). Before pre-clearing, 50 μl of the cleared lysate was removed as input for total RNA profiling. Pre-cleared lysate was incubated with 15 μg of Ago2 antibody (ab57113, Abcam) at 4°C for 3 hours. After antibody incubation, 50 μl of protein-G beads were added to the lysate and incubated for 1 hour at 4°C. Beads were washed 8 times with lysis buffer and Ago2-RNA complexes were extracted by adding 1 ml TRIzol reagent (Invitrogen) directly to the beads. RNA extraction was carried out as per the manufacturers instructions.

### Microarray

Total RNA or RNA isolated from the RISC immunoprecipitations were amplified and biotin-labeled with the Illumina TotalPrep RNA Amplification Kit (Life Technologies). RNA expression was profiled on HumanHT-12 v4 Expression BeadChips (Illumina) at the KECK Biotechnology Resource Laboratory at Yale. Array data can be found in the GEO repository under accession number GSE4616.

### RNA-seq library preparation and sequencing

rRNA was removed using the Ribo-Zero rRNA Removal Kit (Epicentre), and the remaining RNA was purified using the RNeasy MinElute Cleanup Kit (Qiagen). RNA was then fragmented using the NEBNext Magnesium RNA Fragmentation Module (New England BioLabs), and size selected using AMPure XP beads (Agencourt) at 1.8× volume. The ends of the fragmented RNA were then prepared for adaptor ligation using T4 Polynucleotide Kinase (New England BioLabs), and the reaction was cleaned up with the RNeasy MinElute Cleanup Kit (Qiagen). Libraries were then prepared from the now rRNA depleted, fragmented, size selected, and kinased RNAs using the NEBNext Small RNA Library Prep Set according to the manufacturers protocol (New England BioLabs). Libraries were cleaned and further size selected using 0.8× volume AMPure XP beads, and then additional size selections with 1.0× beads were performed as necessary. A minimum of 20 million paired end 100 bp reads were generated for each replicate using a HiSeq 2000 (Illumina) by the Genomic Sequencing and Analysis Facility at the University of Texas at Austin. RNA-seq data can be found in the GEO repository under accession number GSE4616.

### RNA-seq data processing

Barcodes were removed, adaptors were removed using Cutadapt, and reads mapping to rRNAs and tRNAs were filtered. Reads were mapped to the human genome (hg19) by TopHat2 software version 2.0.9 [49]. We combined biological replicates and assigned an expression value (FPKM) for each RefSeq gene with Cuffdiff [49]. To avoid denominator inflation in subsequent ratio calculations, all FPKMs less than 1 were hard capped to 1.

### RNA-seq gene expression repression

Repression of gene expression for each gene was calculated as the ratio of the FPKM in the miRNA transfection to the FPKM in the control transfection:

$$ \mathrm{Repression} = \frac{\mathrm{FPKM}\ \mathrm{f}\mathrm{o}\mathrm{r}\ \mathrm{the}\ \mathrm{control}}{\mathrm{FPKM}\ \mathrm{f}\mathrm{o}\mathrm{r}\ \mathrm{the}\ \mathrm{miRNA}} $$

### RNA-seq RISC immunoprecipitation enrichment

RNA-seq RISC immunoprecipitations (RIP) enrichment was calculated as follows: RIP FPKMs were first normalized to gene expression FPKMs, and then enrichment was calculated as the ratio of the normalized miRNA transfection to the normalized control transfection:

$$ \mathrm{Enrichment} = \frac{\mathrm{FPKM}\ \mathrm{f}\mathrm{o}\mathrm{r}\ \mathrm{the}\ \mathrm{miRNA}\ \mathrm{RIP}\ /\ \mathrm{FPKM}\ \mathrm{f}\mathrm{o}\mathrm{r}\ \mathrm{the}\ \mathrm{miRNA}\ \mathrm{gene}\ \mathrm{expression}}{\mathrm{FPKM}\ \mathrm{f}\mathrm{o}\mathrm{r}\ \mathrm{the}\ \mathrm{control}\ \mathrm{RIP}\ /\ \mathrm{FPKM}\ \mathrm{f}\mathrm{o}\mathrm{r}\ \mathrm{the}\ \mathrm{control}\ \mathrm{gene}\ \mathrm{expression}} $$

### Defining a miRNA target list using RIP enrichment and gene expression repression

Target scores to rank each mRNA were generated by averaging RIP enrichment values and gene expression repression values. mRNAs with a score of 1.75 or greater were designated as the target set.

### Gene ontology analysis

Gene ontology analysis was performed with the GeneCodis online tool [38-40].

### Searching for non-canonical target pairing

The subsequences of miR-503 and miR-103 examined for pairing were selected based off the subsequence enrichment plots shown in Figure 4A. For miR-503, the criteria used to search for compensatory sites (5′ mismatch + additional 3′ pairing) were: (1) a 6-mer pairing position 1–6 with 1 mismatch, (2) an 8-mer pairing position 11–18 with 0 – 3 mismatches, and (3) a 1 base pair wobble allowed in the length of the sequence separating the 6-mer and 8-mer. To search for supplementary sites (5′ perfect pairing + additional 3′ pairing) the criteria used were: (1) a 6-mer perfectly pairing position 1–6, (2) an 8-mer pairing position 11–18 with 0 – 3 mismatches, and (3) a 1 base pair wobble allowed in the length of the sequence separating the 6-mer and 8-mer. A slightly offset seed sequence (position 1–6) was used for miR-503 because it was slightly more enriched in the experimentally identified targets (Figure 4A). For miR-103, the criteria used to search for compensatory sites (5′ mismatch + additional 3′ pairing) were: (1) a 6-mer pairing position 2–7 with 1 mismatch, (2) an 8-mer matching position 11–18 with 0 – 3 mismatches, and (3) a 1 base pair wobble allowed in the length of the sequence separating the 6-mer and 8-mer. To search for supplementary sites (5′ perfect pairing + additional 3′ pairing) the criteria used were: (1) a 6-mer perfectly pairing position 2–7, (2) an 8-mer pairing position 13–20 with 0 – 3 mismatches, and (3) a 1 base pair wobble allowed in the length of the sequence separating the 6-mer and 8-mer.

### Luciferase reporter assays

We cloned 0.9 kb of the 3′UTR of DDHD2 centered around the putative miR-503 binding site into the psi-CHECK2 plasmid (Promega) downstream from the Renilla luciferase gene using XhoI and NotI restriction sites. Forward primer (lower case indicates XhoI site): TTGTTGctcgagTTTTCCTCTCCTCAGCTGCG.

Reverse primer (lower case indicates NotI site): CTCTATgcggccgcGCCTGTGGAATCTGAGAGCA. This plasmid was used to produce three mutant variants. In the whole-site deletion variant, the entire 22 bp sequencing corresponding to the length of miR-503 at its binding site was deleted (QuikChange Lightning Mutagenesis Kit, Agilent). In the 3′ deletion variant, the 6 bp sequence corresponding to positions 7–12 of the putative binding site (Figure 5B) (and positions 11–16 of the miRNA in a putative duplex) was deleted. In the 5′ deletion variant, the 6 bp sequence corresponding to positions 17–22 of the putative binding site (Figure 5B) (and positions 2–7 of the miRNA in a putative duplex) was deleted.

HEK293 cells were plated in 24-well plates at 10^5^ cells/well in DMEM supplemented with 10% FBS and 1% Pen-Strep and grown overnight (Hyclone, Gibco). Each plasmid was co-transfected at 50 ng/well with either miRNA or Control siRNA duplex at a concentration of 100 nM in triplicate (Lipofectamine, Invitrogen). Cells were harvested 24 hours post-transfection and luciferase activity was measured using the Promega Dual Luciferase kit according to the manufacturer’s instructions. Data was first normalized per-well by dividing Renilla luminescence by Firefly luminescence. Relative luciferase activity was then calculated as the ratio of the mean of the three biological replicates for each miRNA transfected group to the mean of the corresponding Control siRNA transfected group.

### Western blotting

Primary human fibroblasts or HeLa cells were seeded in 6-well plates at 5 × 10^4^ cells/well in DMEM supplemented with 10% FBS (Hyclone, Invitrogen). 24 hours after plating, miR-503 or Control siRNA duplexes were transfected at a 100 nM concentration (Lipofectamine, Invitrogen). Transfected cells were lysed at 24 hr, 48 hr, 72 hr, or 96 hr post-transfection.

Cell lysates were separated on 4-20% gradient SDS-PAGE gels (Biorad) and proteins were transferred onto PVDF membranes. Membranes were blocked with 5% milk or 5% BSA in TBST and probed overnight with primary antibody in blocking solution (DDHD2: 1/500, Abcam, ab103965; GAPDH: 1/1000, Abcam, ab9486). The membranes were washed, incubated with HRP-conjugated secondary antibody in blocking solution (1/5000, Santa Cruz Biotechnology, sc-2004), and washed again. HRP substrate solution was added to the membranes, and incubated for four minutes (Pierce). Blots were exposed to autoradiographic film and developed (Carestream Kodak Biomax). Images of the films were scanned and band intensities quantified using a white-light transilluminator imaging system (FlourChem Q, ProteinSimple).

### Cancer survival and copy number alteration analysis

Survival probabilities of ER+ breast cancer patients were generated with MIRUMIR (http://www.bioprofiling.de/GEO/MIRUMIR/mirumir.html) [43]. Data on DDHD2 copy number alteration frequency and patient survival in patients with different cancer types was generated using cBio portal (http://www.cbioportal.org/public-portal/) [50]. For each cancer type the percentage of patients with DDHD2 amplifications, and patient survival probabilities, were calculated as an average of all data sets available for this cancer type. P-values were calculated with a hypergeometric distribution.

### Statistical analysis

Statistical significance was estimated using a one-sided, two sided, or paired Student’s t-test as indicated, assuming unequal variance.

## Additional files

Additional file 1:
**Spearman rank correlation between RNA-seq FPKM and microarray intensity value for each gene profiled by both RNA-seq and FPKM.** The average FPKM and average microarray intensity values of the biological replicates were calculated for all conditions. Values in the table are the Spearman rank correlation coefficients comparing the averaged RNA-seq FPKM and microarray intensity values. Additional file 2:
**GO term enrichment for experimentally determined miRNA targetomes for miR-503 and miR-494.**
Additional file 3:
**Using RIP-ChIP and microarray gene expression profiling to explore miRNA target pairing outside of the canonical miRNA 5′ seed match.** (A) Enrichment of sequences pairing to the 3′ end of the miRNAs in miRNA targetomes constructed by combining RIP-ChIP and microarray expression profiling data. Bars are the frequency indicated on the Y-axis of a 6-mer in the miRNA targetome relative to all mRNAs profiled. 6-mers are organized along the X-axis from 5′ end to 3′ end of the mature miRNA. (B) Frequency of different types of miR-503-target pairing in RIP-ChIP enriched mRNAs. Bars are the frequency of mRNAs with the indicated type of miRNA-target pairing in the RIP enriched mRNAs, relative to the frequency of mRNAs with the indicated type of miRNA pairing in all mRNAs profiled. (C) Left: RIP-ChIP enriched mRNAs containing a complementary target site in their 3′UTR are significantly more repressed than all RIP-ChIP enriched mRNAs, but not more repressed than all RIP-ChIP enriched mRNAs that only contain a perfect 5′ seed match (p = 0.04 and 0.10, respectively, Student’s t-test). Right: There were no significant differences in miR-503 RIP enrichment or gene expression in RIP enriched mRNAs with different types of miRNA pairing. (D) There were no significant differences between different types of miR-103 target pairing in miR-103 RIP-seq enriched mRNAs in either RIP enrichment (left) or gene expression repression (right). For C and D, each box and whiskers indicates gene expression repression for genes that contain the type of miRNA-target pairing specified on the X-axis. Boxes extend from the 1^st^ to 3^rd^ quartile of gene expression repression, the band is the median, and whiskers denote the minimum and maximum excluding outliers. Each plot indicates RIP enrichment or gene expression repression for mRNAs that contain the type of miRNA-target pairing specified on the X-axis.

## Abbreviations

RISC: RNA-induced silencing complex
RIP: RISC immunoprecipitation
RIP-chip: Identifying transcripts isolated from RISC immunoprecipitations using microarrays
RIP-seq: Identifying transcripts isolated from RISC immunoprecipitations using deep sequencing
ER+: Estrogen receptor positive
NSCLC: Non small cell lung cancer
FBS: Fetal bovine serum
LNA: Locked nucleic acid
