# Supplementary material for: miR-503 represses human cell proliferation and directly targets the oncogene DDHD2 by non-canonical target pairing
Source: BMC Genomics. 2015 Feb 5;16(1):40. doi: 10.1186/s12864-015-1279-9 (PMC4326481; doi:10.1186/s12864-015-1279-9)
Supplement: Additional file 1: — Spearman rank correlation between RNA-seq FPKM and microarray intensity value for each gene profiled by both RNA-seq and FPKM. The average FPKM and average microarray intensity values of the biological replicates were calculated for all conditions. Values in the table are the Spearman rank correlation coefficients comparing the averaged RNA-seq FPKM and microarray intensity values. [file 12864_2015_1279_MOESM1_ESM.docx]

Spearman rank correlation between RNA-seq FPKM and microarray intensity value for each gene profiled by both RNA-seq and FPKM.

|  | miR-503 | miR-103 | miR-494 | mock | Control siRNA |
| --- | --- | --- | --- | --- | --- |
| RIP | 0.74 | 0.72 | 0.74 | 0.72 | 0.73 |
| Expression | 0.72 | 0.72 | 0.72 | 0.72 | NA |
