# Supplementary figures and images for: miR-503 represses human cell proliferation and directly targets the oncogene DDHD2 by non-canonical target pairing
Source: BMC Genomics. 2015 Feb 5;16(1):40. doi: 10.1186/s12864-015-1279-9 (PMC4326481; doi:10.1186/s12864-015-1279-9)

A

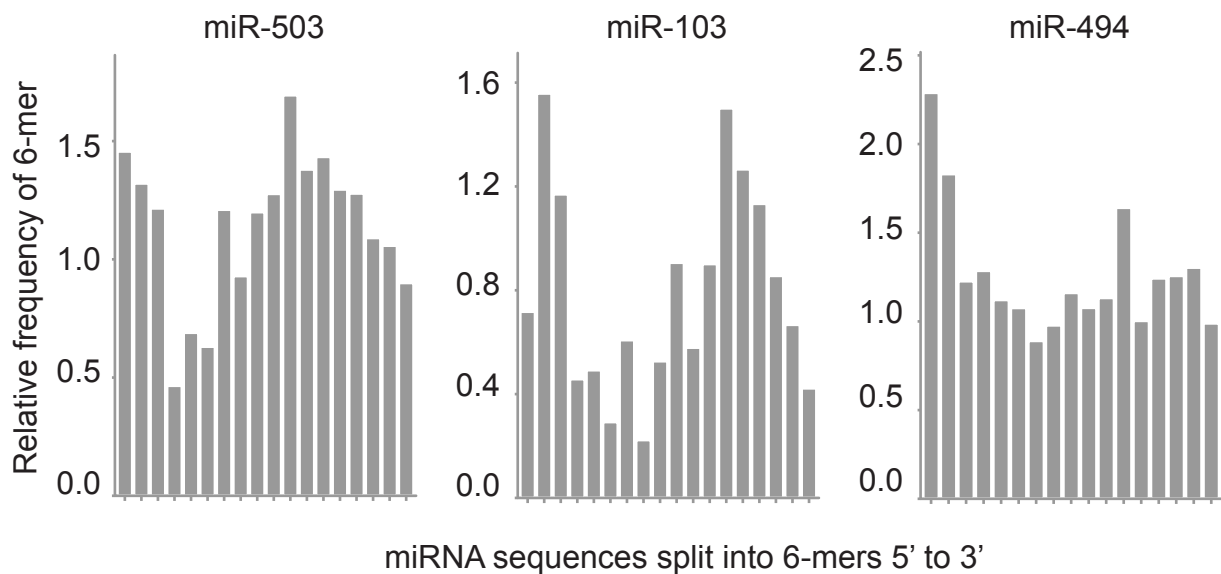

B

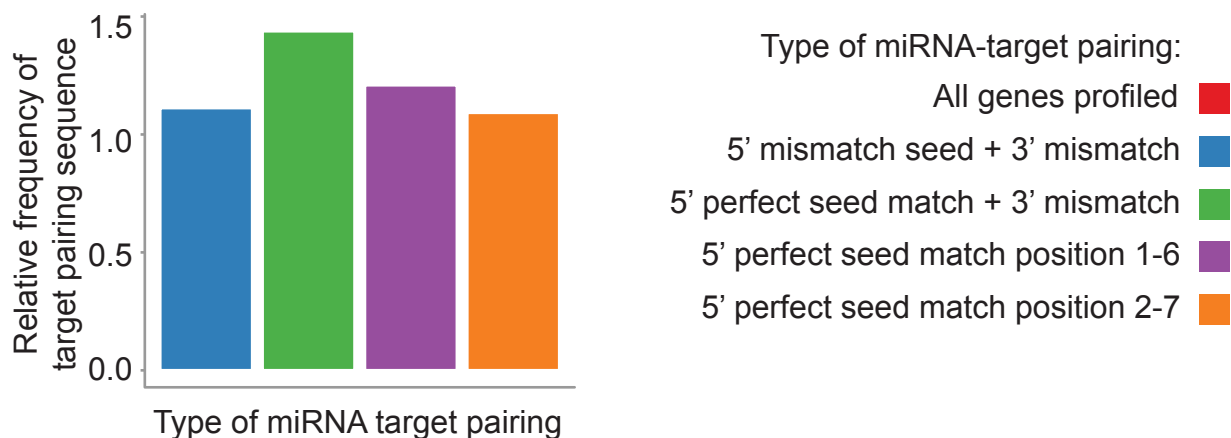

C

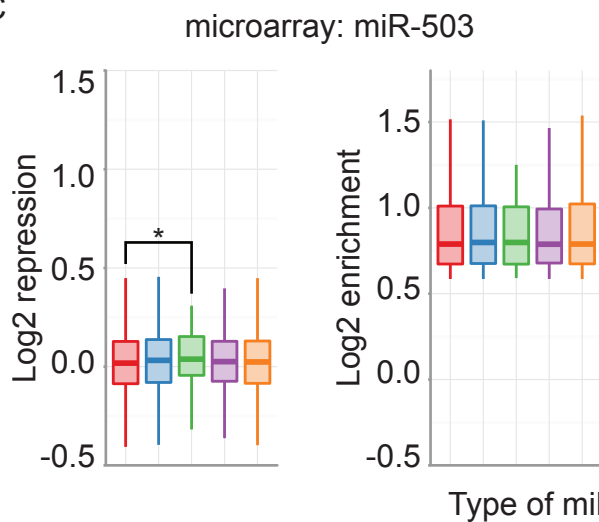

D

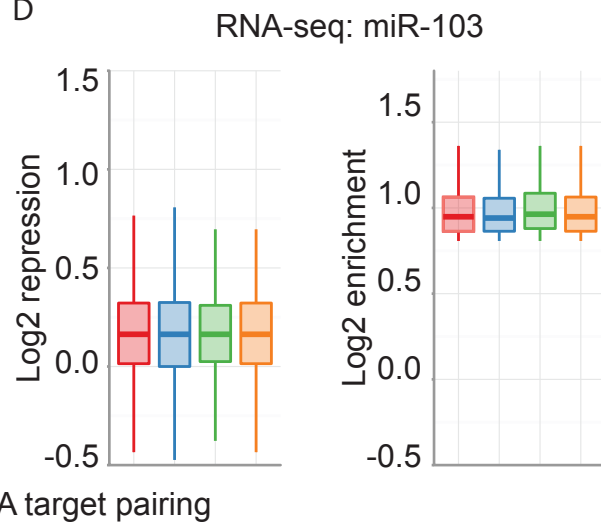

Supplement: Additional file 3: — Using RIP-ChIP and microarray gene expression profiling to explore miRNA target pairing outside of the canonical miRNA 5′ seed match. (A) Enrichment of sequences pairing to the 3′ end of the miRNAs in miRNA targetomes constructed by combining RIP-ChIP and microarray expression profiling data. Bars are the frequency indicated on the Y-axis of a 6-mer in the miRNA targetome relative to all mRNAs profiled. 6-mers are organized along the X-axis from 5′ end to 3′ end of the mature miRNA. (B) Frequency of different types of miR-503-target pairing in RIP-ChIP enriched mRNAs. Bars are the frequency of mRNAs with the indicated type of miRNA-target pairing in the RIP enriched mRNAs, relative to the frequency of mRNAs with the indicated type of miRNA pairing in all mRNAs profiled. (C) Left: RIP-ChIP enriched mRNAs containing a complementary target site in their 3′UTR are significantly more repressed than all RIP-ChIP enriched mRNAs, but not more repressed than all RIP-ChIP enriched mRNAs that only contain a perfect 5′ seed match (p = 0.04 and 0.10, respectively, Student’s t-test). Right: There were no significant differences in miR-503 RIP enrichment or gene expression in RIP enriched mRNAs with different types of miRNA pairing. (D) There were no significant differences between different types of miR-103 target pairing in miR-103 RIP-seq enriched mRNAs in either RIP enrichment (left) or gene expression repression (right). For C and D, each box and whiskers indicates gene expression repression for genes that contain the type of miRNA-target pairing specified on the X-axis. Boxes extend from the 1st to 3rd quartile of gene expression repression, the band is the median, and whiskers denote the minimum and maximum excluding outliers. Each plot indicates RIP enrichment or gene expression repression for mRNAs that contain the type of miRNA-target pairing specified on the X-axis. [file 12864_2015_1279_MOESM3_ESM.pdf]
